# Delayed Biloma Secondary to an Iatrogenic Common Hepatic Duct Injury After Open Cholecystectomy: A Rare Cause of Persistent Biliary Drainage

**DOI:** 10.7759/cureus.94486

**Published:** 2025-10-13

**Authors:** Yoseph M Habte, Binyam M Habte, Makida M Habte, Esimael M Abdu, Biruk W Fantu

**Affiliations:** 1 Department of Medicine, Ethio Tebib Hospital, Addis Ababa, ETH; 2 Department of Medicine, ALERT Comprehensive Specialized Hospital, Addis Ababa, ETH; 3 Department of Medicine, Bethel Medical College, Addis Ababa, ETH; 4 Department of Surgery, Teklehaimanot General Hospital, Addis Ababa, ETH; 5 Department of Medicine, Axon Stroke and Spine Center, Addis Ababa, ETH

**Keywords:** biliary leakage, biloma, case report, common hepatic duct injury, roux-en-y hepaticojejunostomy

## Abstract

Bilomas are localized collections of bile outside the biliary tree, most often resulting from an iatrogenic injury following hepatobiliary procedures. While bile leaks typically arise from the cystic duct stump and present early in the postoperative period, delayed biloma formation due to a proximal bile duct injury is uncommon. We report the case of a 35-year-old female who presented 52 days after open cholecystectomy with persistent bilious wound discharge, right upper quadrant pain, and intermittent jaundice. Abdominal ultrasonography revealed a subhepatic fluid collection communicating with the cystic duct stump, consistent with a biloma. Magnetic resonance cholangiopancreatography (MRCP) confirmed the biloma and demonstrated residual cystic duct calculi along with mild intrahepatic biliary dilatation. When conservative management failed, exploratory laparotomy revealed an iatrogenic injury to the common hepatic duct. A Roux-en-Y hepaticojejunostomy (RYHJ) was performed, leading to clinical improvement and resolution of the bile leak. At her two-week outpatient follow-up, the patient remained asymptomatic with normalized liver function tests. This case underscores the diagnostic challenge of delayed biloma secondary to a proximal bile duct injury and highlights the value of early imaging in guiding timely surgical management.

## Introduction

Bile leaks remain one of the most concerning complications following cholecystectomy, with an incidence reported between 0.8% and 1.1% [1]. While most involve the cystic duct stump or accessory ducts, such as ducts of Luschka, injury to the common hepatic duct (CHD) is uncommon and often associated with significant morbidity [2]. Biloma, defined as an encapsulated bile collection outside the biliary tree, typically arises early in the postoperative period; delayed presentations, particularly several weeks after surgery, are rarely reported [3,4].

Bilomas are most commonly iatrogenic but may also result from spontaneous rupture of the biliary system [5]. They form when leaked bile accumulates in the peritoneal cavity, triggering local inflammation and eventual encapsulation. Symptoms range from vague abdominal discomfort, fever, and malaise to more specific signs such as jaundice or persistent bilious wound drainage [1,5]. These features often overlap with those of intra-abdominal abscesses, particularly when complicated by infection [5].

While ultrasonography and computed tomography (CT) are useful initial imaging tools, magnetic resonance cholangiopancreatography (MRCP) offers superior visualization of biliary anatomy and more accurate leak localization. Management depends on biloma size, leak severity, and the patient’s overall condition [1,5,6]. Small, asymptomatic collections may resolve spontaneously, but larger or infected bilomas typically require percutaneous drainage or endoscopic intervention. Complex or high-grade injuries involving the CHD necessitate surgical repair, such as Roux-en-Y hepaticojejunostomy (RYHJ) to restore biliary continuity and prevent complications like infection, fistula formation, or strictures [1,6].

This case describes a delayed biloma secondary to an iatrogenic CHD injury diagnosed nearly two months post-cholecystectomy, highlighting the diagnostic challenges and need for individualized surgical management in complex biliary injuries.

## Case presentation

A 35-year-old female presented 52 days after undergoing an open cholecystectomy for symptomatic cholelithiasis, with a 1-month history of greenish discharge from the surgical wound site. The discharge had reportedly begun approximately three weeks postoperatively and progressively increased in volume over the two weeks preceding presentation. She also reported mild, dull right upper quadrant abdominal pain, fatigue, decreased appetite, and intermittent yellowing of the eyes. She remained afebrile throughout admission. There was no history of bowel or urinary habit changes, and no prior cardiac or renal disease.

**Figure 1:**
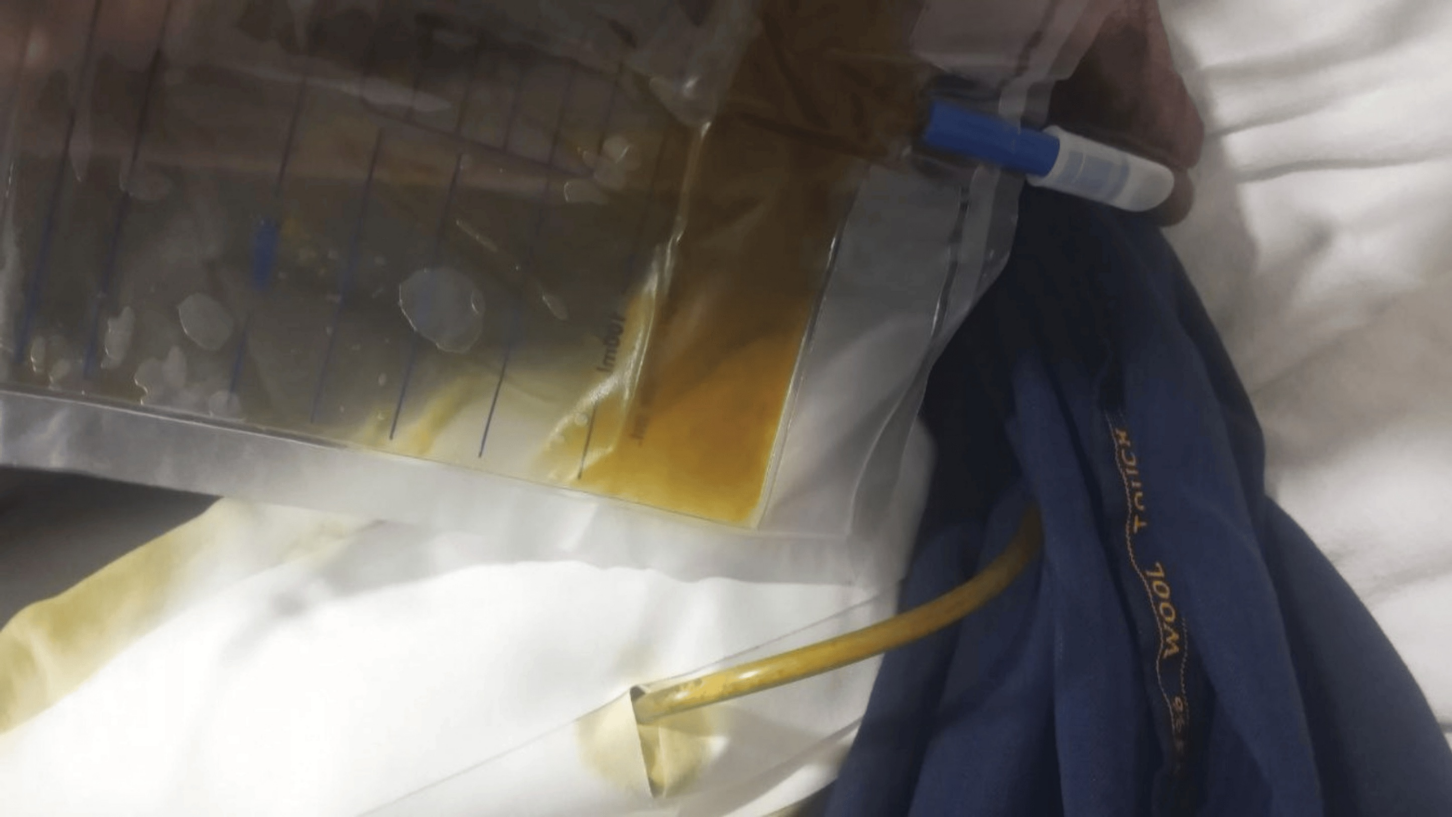
Drainage tube in situ at prior cholecystectomy site with visible bilious output (~100 mL/day)

On physical examination, the patient appeared ill. She was hemodynamically stable. Conjunctival pallor was absent, but mild scleral icterus was noted. The abdomen was distended, with a drainage tube in situ at the previous cholecystectomy site, showing approximately 100 mL of bilious output per day (Figure 1). Mild tenderness was elicited on palpation, but there were no signs of peritonitis or organomegaly. Cardiovascular, respiratory, neurological, and musculoskeletal examinations were unremarkable.

Laboratory investigations revealed elevated liver enzymes (aspartate aminotransferase (AST) 73.1 U/L, alanine aminotransferase (ALT) 49.1 U/L, alkaline phosphatase (ALP) 354 U/L), total bilirubin of 3.11 mg/dL (direct 1.42 mg/dL), and a raised C-reactive protein level of 44.39 mg/L. Coagulation studies showed a prolonged prothrombin time (19.5 seconds) and an elevated international normalized ratio (INR) of 1.60. Serum potassium was low at 2.94 mmol/L. Complete blood count was within normal limits, except for thrombocytosis (472,000/µL) (Table 1).

**Table 1:** Laboratory investigations with corresponding results and reference values

Laboratory Parameters	Results	Normal Value
Complete Blood Count		
White Blood Cell	8.3 × 10^3^/µL	4.0 - 11.0 × 10^3^/µL
Hemoglobin	14.2 g/dL	12.0 - 16.0 g/dL
Platelet	472 × 10^3^/µL	150 - 450 × 10^3^/µL
Lymphocyte Percentage	44.40%	15 - 50%
Neutrophil Percentage	48%	45 - 80%
Metabolic Panel		
Creatinine	0.79 mg/dL	0.51 - 0.95 mg/dL
Urea	28.8 mg/dL	17- 43 mg/dL
Na+	139 mmol/L	136 - 145 mmol/L
K+	2.94 mmol/L	3.5 - 5.1 mmol/L
Aspartate Transaminase	73.1 U/L	2.0 - 35.0 U/L
Alanine Transaminase	49.1 U/L	1 - 35.0 U/L
Alkaline Phosphatase	354 U/L	70 – 260 U/L
Total Bilirubin	3.11 mg/dL	0.3 – 1.2 mg/dL
Direct Bilirubin	1.42 mg/dL	< 0.2 mg/dL
C-Reactive Protein	44.39 mg/L	< 5 mg/L
Procalcitonin	0.41 ng/mL	< 0.05 ng/mL
Coagulation Profile		
Prothrombin Time	19.5 seconds	10.7 - 14.3 seconds
International Normalized Ratio	1.6	0.8 - 1.2
Activated Partial Thromboplastin Time	33.5 seconds	21 - 35 seconds
Serology		
Venereal Disease Research Laboratory Test (VDRL)	Non-reactive	Non-reactive
Hepatitis B Surface Antigen	Non-reactive	Non-reactive
Hepatitis C Antibody	Non-reactive	Non-reactive
Rapid HIV Diagnostic Test	Non-reactive	Non-reactive

Abdominal ultrasonography showed a well-defined, thick-walled hypoechoic fluid collection in the subhepatic region measuring approximately 5.0 × 1.8 × 1.8 cm surrounding the distal end of the drainage tube. The gallbladder was not visualized. There was no intrahepatic ductal dilatation or focal hepatic lesion.

**Figure 2:**
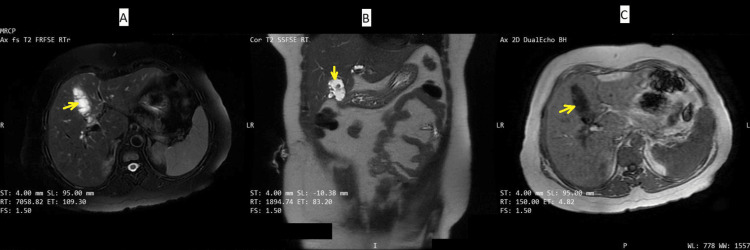
Multiplanar MRCP demonstrating post-cholecystectomy biloma with cystic duct stump communication Multiplanar MRCP imaging demonstrating a 6.4 × 2.5 × 3.4 cm fluid collection in the gallbladder fossa (yellow arrow), appearing: (A) Hyperintense on axial T2-weighted imaging (B) Hyperintense on coronal T2-weighted imaging (C) Hypointense on axial T1-weighted imaging MRCP - Magnetic Resonance Cholangiopancreatography

MRCP demonstrated a 6.4 × 2.5 × 3.4 cm fluid collection in the gallbladder fossa with internal septations and imaging characteristics suggestive of a biloma. The collection appeared to communicate with the cystic duct stump. Mild intrahepatic biliary ductal dilatation was noted, but the common bile duct was of normal caliber without evidence of obstruction. Multiple T2-hypointense filling defects were seen within the cystic duct stump, the largest measuring 8.6 mm, consistent with residual calculi. An incidental hepatic hemangioma was identified in segment VII (Figure 2).

The patient was initially managed with intravenous antibiotics (ceftriaxone 1 g every 12 hours and metronidazole 500 mg every 8 hours). Blood and wound cultures showed no growth. The electrolyte abnormality (hypokalemia) was corrected with intravenous potassium supplementation. A sepsis workup was performed, including measurement of procalcitonin, which was mildly elevated (0.41 ng/mL), but there was no clinical evidence of systemic infection. Given the persistent bilious discharge and imaging findings suggestive of a biliary leak with possible cystic duct or common hepatic duct injury, surgical intervention was planned.

At laparotomy, dense adhesions were encountered between the liver, colon, and duodenum. Intraoperative findings confirmed an iatrogenic injury to the common hepatic duct. The injured duct was opened proximally up to the left hepatic duct. A RYHJ was performed with an end-to-side anastomosis between the left hepatic duct and a prepared jejunal limb. A jejunojejunostomy was completed, and a subhepatic drain was placed. The peritoneal cavity was irrigated, and the abdomen was closed in layers.

The patient tolerated the procedure well and was transferred to the recovery unit in stable condition. Postoperatively, the subhepatic drain initially collected approximately 5 mL of bile over the first 24 hours, with no further leakage observed over the subsequent 48 hours. She was maintained on intravenous antibiotics, gradually advanced to oral intake, and demonstrated clinical and laboratory improvement. Consequently, she was discharged from the hospital after six days of admission in stable condition, with plans for outpatient follow-up. At her two-week outpatient follow-up, she remained asymptomatic, and her liver function tests were within normal limits.

## Discussion

Biloma is defined as a loculated collection of bile outside the biliary tree within the abdominal cavity [1-3]. It results primarily from iatrogenic injury, trauma, or spontaneous rupture of the biliary system [2]. Postoperative bile leak after cholecystectomy occurs in approximately 0.8%-1.1% of cases, with the cystic duct stump being the most common site of leakage. Bile leaks are classified into low-grade and high-grade types based on the site of biliary injury, with high-grade leaks typically involving major bile ducts and presenting early within the first week postoperatively [4]. In this case, despite the high-grade nature of the iatrogenic common hepatic duct injury, the patient presented with a delayed biliary leak and subsequent biloma formation.

The pathogenesis of biloma depends on the rate and site of bile leakage and the rate of peritoneal absorption [1]. Rapid extravasation typically leads to encapsulated collections and localized inflammation, whereas slower leaks cause mild peritoneal irritation without generalized peritonitis [1,7]. Imaging revealed a well-defined fluid collection communicating with the cystic duct stump, along with mild intrahepatic biliary dilatation, consistent with bile duct injury and biloma formation. Residual calculi within the cystic duct likely increased biliary pressure, perpetuating the leak.

Clinically, bilomas present variably with abdominal pain, fever, jaundice, or persistent bilious drainage from surgical wounds or drains [7,8]. Laboratory abnormalities, such as elevated liver enzymes, hyperbilirubinemia, and elevated inflammatory markers, may assist diagnosis, but imaging modalities, including ultrasonography, CT, and MRCP, are essential for accurate localization and characterization of the bile collection [1,8]. Our case utilized MRCP effectively to define the biloma and its biliary communication, which guided management.

Treatment depends on biloma size, bile leak severity, and clinical status. Current practice favors less invasive options where possible [1,4]. ERCP with sphincterotomy and transpapillary stenting is first-line for most bile leaks, promoting bile flow through the papilla, reducing the transpapillary pressure gradient, and facilitating leak healing [4,8,9]. Importantly, the cholangiographic distinction between low-grade and high-grade bile leaks is instrumental in directing appropriate endoscopic management [4].

When endoscopic intervention is unsuccessful or not feasible, percutaneous drainage and embolization techniques offer effective alternatives [10]. Potential complications of minimally invasive approaches include infection, ductal perforation, hemorrhage, and device displacement, highlighting the need for careful patient selection [11].

Definitive surgical repair, such as RYHJ, is required for persistent or complex bile duct injuries, especially involving the common hepatic duct, as in our patient [1,12]. Although risks of postoperative complications exist, including anastomotic strictures or leaks, refined surgical techniques have reduced morbidity, especially with minimally invasive approaches [12]. Our patient’s persistent bilious drainage and confirmed common hepatic duct injury warranted RYHJ, resulting in clinical improvement and resolution of the biloma.

In conclusion, this case highlights the importance of timely diagnosis and individualized management of postoperative bile leak and biloma, based on the severity of injury and the patient's clinical condition. Early recognition through appropriate imaging, along with a multidisciplinary treatment approach encompassing endoscopic, percutaneous, and surgical options, is vital to prevent morbidity and optimize patient outcomes.

This report describes a single patient, which limits the generalizability of the findings. Certain diagnostic and follow-up data were limited to what was clinically indicated. Nevertheless, this case provides valuable clinical insights into the recognition and management of delayed biloma and high-grade bile duct injuries, highlighting lessons applicable to similar clinical scenarios.

## Conclusions

Delayed biloma formation secondary to an iatrogenic common hepatic duct injury is a rare but important differential in patients presenting with persistent postoperative biliary drainage. High clinical suspicion, appropriate imaging, and timely surgical intervention are critical for favorable outcomes. This case reinforces the diagnostic value of MRCP in complex biliary presentations and highlights the role of RYHJ in definitive management when conservative measures fail. Awareness of such atypical postoperative complications is essential to minimize morbidity and guide optimal care.
